# Multiple consecutive sphincter-preserving procedures improve overall healing outcomes in transsphincteric fistulae: a prospective cohort study of 101 patients

**DOI:** 10.1007/s10151-026-03352-2

**Published:** 2026-05-15

**Authors:** E. Anand, A. Senapati

**Affiliations:** 1 St Mark’s The National Bowel Hospital, Acton Lane, London, UK; 2 Imperial College London, London, UK; 3 Portsmouth Hospitals University NHS Trust, Portsmouth, UK

**Keywords:** Perianal fistula, Transsphincteric fistula, Fistula plug, LIFT, Advancement flap

## Abstract

**Background:**

The management of complex transsphincteric fistulae is challenging and surgical repair must be balanced against the risk of continence impairment. This study aims to demonstrate that multiple, carefully selected procedures can achieve long-term clinical healing without sphincter division for the majority of patients in this cohort.

**Methods:**

This was a prospective cohort study in a large university hospital with a dedicated colorectal surgery unit. Patients underwent between one and four sphincter-preserving procedures, including advancement flap (AF), ligation of the intersphincteric fistula tract (LIFT), and fistula plug placement. The primary outcome was overall clinical healing at the end of the study period. Secondary outcomes included the incidence of incontinence and the success rates of individual sphincter-preserving procedures.

**Results:**

Out of 120 patients, 101 were eligible for final analysis. The mean patient age was 43.3 years, with a median follow-up duration of 21 months (IQR 11–31). The overall clinical healing rate was 90.1% (91/101), achieved after a mean of 1.6 procedures (SD 0.85, Range 1–4). Among patients who did not undergo sphincter division, the healing rate was 88.4% (76/86). The advancement flap demonstrated the highest success rate (85.7%), compared with LIFT (56%) and plug (38.8%) as first-stage procedures, with a statistically significant difference (*p* = 0.007).

**Conclusions:**

Complex transsphincteric fistulae can be successfully treated with a 90.1% success rate using a combination of sphincter-preserving surgical procedures. Patients should be informed of the likelihood of multiple operations to achieve long-term healing.

## Introduction

### Background

Transsphincteric fistula-in-ano (TSF), classified by Parks et al. in 1961, is a complex anal fistula often originating from an acute anorectal abscess [1]. High transsphincteric fistulae are challenging to manage owing to complex anatomy and up to a 50% risk of continence impairment after sphincter-preserving procedures [2, 3]. Fistulotomy for high TSF carries a high risk of incontinence [4], and owing to limited evidence and high incontinence rate, fistulotomy with immediate sphincter reconstruction (FISR) is rarely performed [5]. This has led to the development of an armamentarium of “sphincter-preserving techniques” such as advancement flaps (AF), ligation of intersphincteric fistula tract (LIFT), video-assisted anal fistula treatment (VAAFT), and laser ablation of fistula tract (LAFT), which have variable (but disappointing) rates of healing and a significant risk of complications including continence impairment [3, 6–9].

The European Society of Coloproctology (ESCP) guidelines and management algorithm highlight the role of sphincter-preserving procedures for cryptoglandular fistulae [10]. LIFT is recommended for suitable fistulae, whereas AF is preferred for high anal fistulae and TSF unsuitable for LIFT. However, meta-analyses have consistently showed that no single approach is superior, and all have limited long-term efficacy [6, 11–14]. Furthermore, the risk of incontinence is not insignificant, with a recent study suggesting up to 50% patients report continence impairment post-LIFT [3] and 77% post-AF [15]. Alternatives including fistula plugs have limited supporting evidence [16–18] although are inherently associated with lower risks of continence impairment and newer techniques such as VAAFT and LAFT carry very low evidence levels but are nonetheless recommended within this algorithm [10, 19, 20]. Managing TSF effectively is essential to minimize morbidity, though existing studies are hindered by small sample sizes, inadequate follow-up, and inconsistent outcome measures [21].

Despite LIFT being recommended as the first-line treatment for straight TSF amenable to repair [10], a recent Dutch retrospective study has suggested that primary fistula healing rates were less than 1 in 3 following LIFT [3] and around 40% following AF [15]. A large multicenter study by Sugrue et al. reported a 44% healing rate for sphincter-preserving procedures for TSF with a median follow-up of 9 months [22]. No single technique has proven superior: surgical success depends heavily on surgeon expertise and prior interventions, with real-world healing rates rarely breaching 50%. This highlights the need for a paradigm shift towards a multi-step treatment strategy rather than reliance on a single intervention.

### Objectives

The primary objective of this study is to demonstrate that multiple, carefully selected procedures are necessary to achieve long-term clinical healing in this challenging cohort of patients. Secondary aims include recording incontinence incidence, the success rates of individual sphincter-preserving procedures, and the number of procedures required to achieve healing.

## Methodology

### Study design

This analysis of a prospectively maintained database evaluated the effectiveness of a definitive sphincter preserving sequential treatment pathway for cryptoglandular transsphincteric fistulae. All patients were managed by a single experienced proctology surgeon (A.S.) and followed for an average of nearly 2 years, with at least two postoperative assessments. The cohort included patients undergoing various sphincter preserving procedures, including fistula plugs, LAFT or FiLaC, LIFT, and AF, while snug setons and lay open procedures were grouped together. Study reporting adhered to STROBE guidelines [11].

### Setting

This study was conducted as a single-centre, prospective cohort study at a large university hospital in the UK. Data were derived from a prospectively maintained fistula database comprising consecutive patients who underwent attempted curative surgery for cryptoglandular TSF. Information on prior fistula surgeries was extracted from electronic medical records at the time of inclusion. Data entry and outcomes were recorded prospectively using a prespecified excel database over the study period and verified independently through chart review (E.A.).

### Participants

Patients included in this study were those diagnosed with cryptoglandular TSF on clinical examination and magnetic resonance imaging (MRI) scan, and who met the following inclusion and exclusion criteria:

### Inclusion and exclusion criteria

The study included adult patients (> 18 years) with cryptoglandular transsphincteric fistulae (the majority were high fistulae), all of whom underwent surgery by a single experienced surgeon. Patients were excluded if they were lost to follow-up despite telephone contact attempts, subsequently diagnosed with perianal Crohn’s disease, declined surgery, did not undergo a definitive sphincter-preserving procedure, or were still undergoing treatment without sufficient follow-up to assess outcomes.

### Outcomes

The primary outcome was clinical healing, defined as the absence of fistula drainage on at least two consecutive clinic visits. Secondary outcomes included the average number of procedures required for healing, success rates of sphincter-preserving surgery at different procedural stages, patient-reported outcomes such as postoperative incontinence and satisfaction (noting this was prior to the validated anal fistula QoL scale), and the development of a treatment pathway flowchart for transsphincteric fistulae.

Patients were stratified into different cohorts according to the type of treatment received and subgroup analysis to compare healing rates and need for further surgery.

### Statistical methods

Data were analyzed using IBM SPSS Statistics (v29). Demographics were summarized as mean ± SD or median (IQR) depending on distribution. Data missing completely at random were excluded from analyses under the assumption that their absence does not introduce bias, and extended telephone follow-up was used to minimize attrition bias. Healing rates were compared using chi-square or Fisher’s exact tests, while multinomial logistic regression identified confounders and predictors. Subgroup analyses comparing success rates across different sphincter-preserving procedures were also performed using chi-square tests.

### Ethical considerations

This project was registered as a service evaluation with the local audit department, ensuring compliance with ethical standards and governance requirements.

## Results

### Participants

**Fig. 1:**
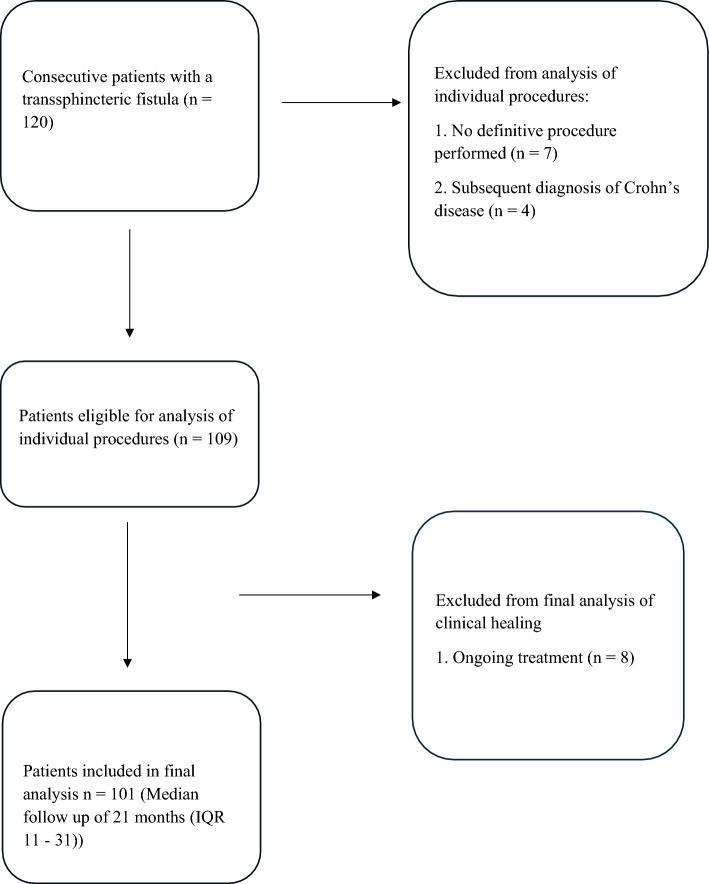
Study flow chart

Of 120 consecutive patients diagnosed with transsphincteric fistulae, 101 patients were deemed eligible for inclusion in our final analysis (Fig. 1). Reasons for exclusion from the study were as follows: no definitive sphincter-preserving procedure performed either because of patient preference or unsuitability for SPP (*n* = 7), subsequent diagnosis of Crohn’s disease (*n* = 4), and ongoing treatment with insufficient follow up to determine healing (*n* = 8).

### Descriptive data

From the initial cohort of 120 patients, 101 were eligible for final analysis (Table 1). The mean age of these patients was 43.3 years (SD 12.2, range 20–73 years), with 51/101 (50.5%) being female. Once missing data was accounted for, the majority of patients were nonsmokers (57.6%) and 73% were classified as ASA Grade 2. Patients had undergone a mean of 3 (SD 3.6) prior nonreparative fistula procedures, including drainage and seton placement, before referral and inclusion in this database.

**Table 1:** Patient demographics

		Median (IQR)	Count (*n*)	Percentage (%)
Age		42 (35–52)		
Sex	Male		50	49.5%
	Female		51	50.5%
ASA grade	ASA I		14	15.6%
	ASA II		66	73.3%
	ASA III		10	11.1%
Smoking status	Nonsmoker		35	58.3%
	Current smoker		18	30.0%
	Ex-smoker Unknown		7 41	11.7%

The median length of follow-up period following the last definitive SPP was 21 months (IQR 11–31), during which patients underwent an average of 3.6 postoperative clinical assessments to determine healing status (range 1–9). Of the 101 patients included in the final analysis, 82 underwent MRI scans, each averaging two scans each during the study period, primarily in the preoperative phase. Initial MRI scans showed that 3% of patients had horseshoeing of the fistula and 4% had abscesses.

The most frequently performed first-stage operation was the fistula plug (*n* = 49, 48.5%), followed by LIFT (*n* = 25, 24.8%) and AF (*n* = 14, 13.9%). For second-stage procedures, LIFT was the most common (28/39, 71.8%). Of 101 patients, 39/101 (38.6%) required two operations, 10/101 (9.9%) had three operations and 6/101 (5.9%) had four operations. A distribution of operative choice across the four stages is provided in Table 2**.**

**Table 2:** Stratification into stages of treatment

Distribution of operative choice across the four stages		
First definitive operation (Total number = 101)		
	*N*	%
Plug	49	48.5%
LIFT	25	24.8%
Advancement flap	14	13.9%
Snug seton/lay open of fistula	11	10.9%
FILAC	2	2.0%

Second definitive operation (Total number = 39**)**		
	*N*	%
LIFT	28	71.8%
Plug	6	15.4%
Advancement flap	2	5.1%
Lay open of fistula snug seton	2 1	5.1% 2.6%

Third definitive operation (Total number = 10)		
	*N*	%
Plug	4	40%
Advancement flap	5	50%
FILAC	1	10%

Fourth definitive operation (*n* = 6)		
	*N*	%
Advancement flap	1	1.0%
Lay open of fistula	1	1.0%
LIFT	2	2.0%
De-epithelialization and primary repair of defect	1	1.0%
Delorme	1	1.0%

A total of 62 (61.4%) patients did not require a second operation. 92 (91.1%) did not require a third operation. A total of 95 (94.1%) did not require a fourth operation

### Outcome data

The overall clinical healing rate in this cohort was 90.1% (91/101) with an average of nearly 2 years of clinical follow-up (Table 3). The overall cumulative clinical healing rate at each stage of procedure increased from 55.4% to 90.1% by the 4th stage procedure (where required). The cumulative healing rate illustrates the incremental benefit of further procedures for the subset of patients who did not respond to initial treatment. The average (mean) number of procedures required to achieve a clinically healed state was 1.6 (SD = 0.9, range 1–4). 56 patients healed after one attempt. Of the remaining 45 patients who required more than one reparative procedure, 35/45 (78%) achieved fistula healing.

**Table 3:** Overall clinically healed

Cumulative healed rate for all transsphincteric fistulas included in the database (*n* = 101)		
Clinically healed rate		
	Number	%
Healed	91	90.1%
Not healed	10	9.9%

Clinically healed rate of patients with high transsphincteric fistulas who did not undergo sphincter division (*n* = 86)		
	Number	%
Healed	76	88.4%
Not healed	10	11.6%

**Fig. 2:**
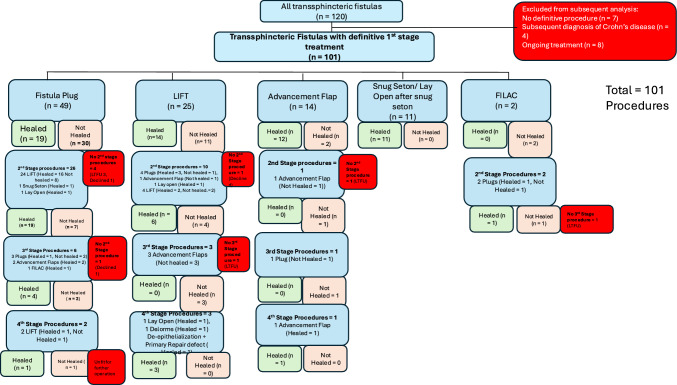
Decision tree pathway

Radiological assessment of healing was not considered standard of care in 87.1% patients unless there was clinical uncertainty, or the patient was part of a trial. Therefore it was not included as a composite endpoint. The outcomes of all 120 patients are shown in Fig. 2.

A total of 15 patients underwent lay open of their fistula due to minimal external sphincter involvement. When these cases are excluded from analysis, 76/86 (88.4%) patients with high TSF achieved clinical healing without sphincter division.

### Complications

Postoperative symptoms of any incontinence were reported in 14/92 (13.9%) (Table 4). Fecal incontinence occurred in four (4.3%), three (3.3%) patients reported occasional anal discharge, two (2.3%) experienced flatus incontinence, four (4.3%) reported leakage, and one (1.1%) had stress incontinence.

**Table 4:** Complications

Incontinence		
	Number	Percent (%)
No incontinence	78	84.8
Symptoms of incontinence	14	13.9
Total	92	100.0
Total	101	

Breakdown of incontinence by procedure type					
Overall Incontinence rate by four common first-stage procedures					
	Plug (*n* = 48)	Snug seton (*n* = 7)		Advancement flap (*n* = 10)	LIFT (*n* = 23)
No incontinence	42 (87.5%)	4 (57.1%)	10 (100%)		18 (78.3%)
Incontinent	2 (4.2%)	0	0		2 (8.7%)
Discharge from anus	1 (2.1%)	2(28.6%)	0		0
Flatus incontinence	1 (2.1%)	0	0		1 (4.3%)
Leakage from anus	2 (4.2%)	0	0		2 (4.2%)
Stress incontinence	0	1 (14.3%)	0		

Two patients in the entire cohort required a stoma during their treatment pathway. Both healed and subsequently had their stomas reversed

### Reasons for not healing

Of the 101 patients included in the final analysis, 91 (90.1%) achieved clinical healing. Ten patients did not achieve healing within the study period. Three patients declined further surgical intervention, and one patient was deemed medically unfit for another general anaesthetic. A further six patients were deemed lost to follow-up, and could not be contacted despite attempts to contact them after the study period had ended. Eight additional patients were excluded from the final analysis because they were still undergoing treatment at the time of data collection. Using multiple imputation to account for missing data, a multinomial logistic regression model was applied to identify potential confounding factors for clinical healing, but no statistically significant variables predictive of healing or complications could be found.

### Subgroup analysis

A subgroup analysis of individual procedural success rate after each stage was performed to elucidate any differences between techniques. After one operation (first stage) there was a statistically significant difference in techniques: AF was successful in 85.7% cases, LIFT in 56% cases, plug in 38.8% (*p* = 0.007). Thereafter, there were no statistically significant differences in treatment rates between the procedures. Across the entire cohort, there was a statistically significant difference across the three main sphincter-preserving procedures (*p* = 0.032). A total of 59 fistula plug operations were performed with a success rate of 40.7% (24/59), 51 LIFT operations were performed with a success rate of 60.8% (31/51), and 22 advancement flaps were performed with a success rate of 68.2% (15/22) (Table 5).

**Table 5:** Subgroup analysis by top three sphincter-preserving procedures

a. Number clinically healed at first attempt by first definitive procedure (% healing rate) (Total number of patients = 101)			
Total number healed	Total number not healed	% Healing rate	*p* value
19	30	38.8	
14	11	56	
12	2	85.7	0.007

b. Overall clinical healed rate for top three sphincter-preserving procedures performed across the entire cohort (Total number of patients = 101)					
	Total number of procedures	Total number healed	Total number not healed	% Healing rate	*p* value
Plug	59	24	35	40.7	
LIFT	51	31	20	60.8	
Advancement flap	22	15	7	68.2	0.032

## Discussion

### Healing rates and follow-up

In this prospective cohort study, we evaluated 120 patients with cryptoglandular TSF using a sphincter-preserving sequential treatment pathway (Fig. 2). The cumulative healing rate of 90.1% was achieved after an average of 1.6 procedures, which is a substantial improvement over the 28–75% healing rate commonly reported in the literature for single-stage AF and LIFT [3, 11, 15, 23]. Once the cases of lay open of low-lying fistulae were excluded from the analysis, an 88.4% rate of definitive healing was achieved without division of sphincters. Our cohort had a median follow-up of 21 months, significantly longer than most randomized controlled trials and large cohort studies, allowing for an average of 3.6 clinical assessments per patient. This extended follow-up enabled us to capture long-term outcomes with greater accuracy and provide a comprehensive evaluation of clinical responses.

This study introduces an alternative approach to evaluate treatment success for TSF, shifting from focusing on individual standalone procedures to a more holistic approach. Both patient and clinician goals for curing a fistula should acknowledge that multiple procedures are often necessary to achieve a satisfactory outcome. Nonetheless, in 10% patients, healing after definitive treatments proved elusive, given the current limitations of available treatments. Whilst the ESCP algorithm should be applauded for bringing clarity to the field, guidelines are based on limited, low-quality evidence (predominantly observational studies) with hugely variable reported success rates and rates of incontinence [3, 10, 15]. Technical expertise in higher risk procedures such as LIFT and AF is diminishing. The absence of dedicated proctology fellowships, restricted tertiary referral pathways for complex perianal fistulae, and resulting low case volumes has lead to limited experience in both LIFT and AF surgery and illustrates the need for a more nuanced shared decision-making approach. We propose a step-up strategy in which patients are first offered lower risk options (that also require less specialist expertise) such as fistula plug, VAAFT, or LAFT, before considering higher risk sphincter preserving procedures. Advancement flap had a higher success rate when performed as a first-line procedure (85.7%) compared with LIFT (56%) or fistula plug (38.8%, *p* = 0.007), although subsequent procedures showed no significant differences. These results must be interpreted in the context of small numbers and the higher operative burden and risk of continence impairment associated with AF [15]. Shared, individualized decision-making remains essential, supporting a step-up approach that reserves higher-risk interventions for patients who do not respond to lower-risk procedures. Many patients may require more than one procedure to achieve healing, and this information should be communicated to patients during shared decision-making.

Although LIFT and AF are reported to carry up to a 50%–80% risk of continence impairment in some series [3, 15], they nonetheless are likely to carry a lower risk of incontinence compared with fistulotomy. During the study period, variation in surgeon preference, experience with newer techniques (which were not available or widely used at the start of the study) meant that not all patients underwent a lower-risk procedure as a first-line option. Nevertheless, minimally invasive techniques such as the fistula plug, VAAFT, or FiLaC (with shorter learning curves) remain reasonable first-line approaches, offering acceptable success rates while minimizing the risk of incontinence. Furthermore not all transsphincteric fistulae may be suitable for certain procedures. This iterative approach reflects real-world clinical practice, where treatment is tailored to patient-specific factors, procedural expertise, and resource considerations, rather than a rigid, predefined algorithm.

### Quality of life and continence

Quality of life, particularly in relation to symptoms and continence, was a key focus of our study **(**Table 4**)**. We observed a low incidence of incontinence, with only 13.9% of patients reporting symptoms of incontinence, which was often subjective and partial. This suggests that our treatment pathway not only effectively heals fistulae but also preserves sphincter function through a step-up approach (whereby patients are typically offered less invasive options initially), which is essential for maintaining patient quality of life. It is, however, likely that continence was better preserved in patients who achieved healing without sphincter division.

These figures are in line with reported rates of incontinence across several meta-analyses [12, 14, 23], although recent evidence has suggested that rates of continence impairment after AF and LIFT may be much higher than initially reported in the literature at between 50 and 77% [3, 15]. Whilst continence was not formally assessed using validated scoring systems, there is ongoing debate about how best to evaluate the different dimensions of continence at an individual patient level rather than relying solely on composite scores [24]. Furthermore, ongoing research in the development of a core outcome measurement set for cryptoglandular fistula, perianal Crohn’s fistulae, and pouch-related fistulae have suggested that there is an unmet need for a fistula-specific continence tool that adequately addresses symptoms originating from the fistula tract itself rather than the anal canal [25].

We compared the success rates of different SPPs within the treatment pathway, specifically the fistula plug, AF, and LIFT. Across the cohort, there was a statistically significant difference in success rates among the three main sphincter-preserving procedures. Overall, fistula plug operations demonstrated a success rate of 40.7%, LIFT procedures achieved a success rate of 60.8%, and AF procedures had the highest success rate at 68.2%. A subgroup analysis of first-stage procedures revealed a statistically significant superiority of the advancement flap procedure, with a healing rate of 85.7%, compared with LIFT at 56% and the fistula plug at 38.8%. Findings are in line with the variation in success reported in the literature for fistula plug (reported healing rates of 54% [16, 17] with low reported incontinence rates [16]), AF (from 43% [15] to 75% [23]), and LIFT (from 28% [3] to 69% [23]). In our cohort, the choice of procedure was influenced by surgeon preference and experience (with experience in LIFT developed midway through the study period), as well as patient choice regarding the associated risks (fistula plug was typically offered as a first-line, low risk procedure). This underscores the importance of individualized treatment plans tailored to each patient’s unique circumstances.

### Limitations

Our study has several limitations. Firstly, the choice of surgical procedure was often influenced by the surgeon’s experience, introducing potential bias. Over the study period, procedural choices evolved as familiarity and expertise with plug, LIFT and AF procedures increased. In addition, as the plug is a noninvasive technique, there was a bias towards offering this in the first instance. VAAFT was not available at our institution during the study period, and LAFT was performed in only two patients late in the study. Although we did not present outcomes for these procedures in our cohort, data from tertiary centers suggest favorable outcomes with low risk of continence impairment, supporting their consideration as low-risk, sphincter-preserving options in other settings [19, 20, 26]. Secondly, as a single-centre, retrospective analysis, our findings may not be entirely generalizable. However, the patient population is representative of those typically seen in general surgical and coloproctology clinics nationwide. We were unable to control for selection bias, as many patients were referred by colleagues with less experience in managing complex fistulae, making them potentially more difficult to treat and achieve clinical healing. Although we addressed attrition bias by ensuring long-term follow-up, it remains a possible issue. Additionally, our decision treatment pathway was influenced by varying levels of surgical experience, available techniques, and technical resources. Consistent with the literature, outcome recording was limited to key outcomes, notably lacking a core outcome set and quality of life monitoring. We did not use a formal quality of life scale nor incontinence score in our study: the use of the recently published cryptoglandular core outcome set and fistula-specific cryptoglandular quality of life scale would greatly enhance future research in this area [21, 27].

### Interpretation

Our study highlights the significance of proper training, and a diverse skill set in managing TSF. Although published data should be interpreted cautiously, given the heterogeneity and limited sample sizes of existing studies, reported rates of postoperative incontinence following advancement flap repair reinforce the importance of individualized, sphincter-preserving treatment selection, with the fistula plug remaining a reasonable option in appropriately selected patients. Lower-risk procedures such as fistula plugs may only offer success rates of around 50% but have a minimal risk of postoperative incontinence and therefore may be preferred by some patients. Higher-risk but potentially higher-reward procedures such as LIFT and AF can results in substantial impairment of continence and may severely affect patients’ quality of life; therefore should be used selectively. Patients should be informed about the potential need for multiple procedures, ranging from one to four, to achieve a 90.1% healing rate. Despite the growing number of available treatments, their success rates and supporting evidence vary. Success rates of individual procedures are consistent with existing literature, which reports success rates between 40 and 75%, often with limited follow-up data [3, 6, 12, 15, 23]. Notably, almost no studies, to our knowledge, have evaluated cumulative healing rates following multiple procedures over a prolonged prospective follow-up, which may more accurately reflect real-world clinical practice. Although the risk of subjectively-reported incontinence in our study was low, this should be viewed in light of our sample size and the rates reported in other studies.

### Generalizability and conclusions

Our findings are broadly applicable to a large district community population, though the single-surgeon experience does limit external validity. We can inform patients of a 90.1% healing rate achieved after one to four procedures, with 84.8% experiencing no continence issues during long-term postoperative follow-up. It is important to emphasize to patients both the benefits and, at times, the necessity of undergoing multiple definitive procedures to achieve long-term clinical healing. This study demonstrates the effectiveness of a sequential treatment pathway for cryptoglandular transsphincteric fistulae without sphincter division in the majority and highlights the importance of personalized patient care to optimize outcomes.

## Data Availability

The datasets generated and/or analyzed during the current study are available from the corresponding author on reasonable request.
